# Na^+^, K^+^-ATPase Subunit Composition in a Human Chondrocyte Cell Line; Evidence for the Presence of α1, α3, β1, β2 and β3 Isoforms

**DOI:** 10.3390/ijms13045019

**Published:** 2012-04-20

**Authors:** Ali Mobasheri, Elisa Trujillo, Mari-Francis Arteaga, Pablo Martín-Vasallo

**Affiliations:** 1 Musculoskeletal Research Group, School of Veterinary Medicine and Science, Faculty of Medicine and Health Sciences, The University of Nottingham, Sutton Bonington LE12 5RD, UK; 2 Rheumatology Service, University Hospital of the Canary Islands, Tenerife 38320, Spain; E-Mail: elisatm@telefonica.net; 3 Developmental Biology Laboratory, Department of Biochemistry and Molecular Biology, University of La Laguna, La Laguna, Tenerife 38206, Spain; E-Mails: marifrancis.arteaga@gmail.com (M.-F.A.); pmartin@ull.es (P.M.-V.)

**Keywords:** Na^+^, K^+^-ATPase, subunit, isoform, isozyme, chondrocyte, cell line, C-20/A4, western blotting, immunofluorescence, FACS analysis

## Abstract

Membrane transport systems participate in fundamental activities such as cell cycle control, proliferation, survival, volume regulation, pH maintenance and regulation of extracellular matrix synthesis. Multiple isoforms of Na^+^, K^+^-ATPase are expressed in primary chondrocytes. Some of these isoforms have previously been reported to be expressed exclusively in electrically excitable cells (*i.e.*, cardiomyocytes and neurons). Studying the distribution of Na^+^, K^+^-ATPase isoforms in chondrocytes makes it possible to document the diversity of isozyme pairing and to clarify issues concerning Na^+^, K^+^-ATPase isoform abundance and the physiological relevance of their expression. In this study, we investigated the expression of Na^+^, K^+^-ATPase in a human chondrocyte cell line (C-20/A4) using a combination of immunological and biochemical techniques. A panel of well-characterized antibodies revealed abundant expression of the α1, β1 and β2 isoforms. Western blot analysis of plasma membranes confirmed the above findings. Na^+^, K^+^-ATPase consists of multiple isozyme variants that endow chondrocytes with additional homeostatic control capabilities. In terms of Na^+^, K^+^-ATPase expression, the C-20/A4 cell line is phenotypically similar to primary and *in situ* chondrocytes. However, unlike freshly isolated chondrocytes, C-20/A4 cells are an easily accessible and convenient *in vitro* model for the study of Na^+^, K^+^-ATPase expression and regulation in chondrocytes.

## 1. Introduction

Chondrocytes are specialized resident cells of articular cartilage responsible for the maintenance and turnover of highly charged extracellular matrix (ECM) macromolecules that endow cartilage with its unique load bearing properties [1,2]. Chondrocytes must survive in an unusual ionic and osmotic environment that makes the maintenance of intracellular [Na^+^], [K^+^] and pH a high priority if the physiological turnover of cartilage matrix is to be accomplished [3]. Membrane transport in cartilage has remained relatively unexplored compared to other cells types. However, over the last decade we have witnessed increasing progress in research aimed at identifying and characterizing ion channels [4], nutrient transporters [5,6] and other types of membrane transporters [3] in chondrocytes. The importance of expanded research into ion and metabolite transport in chondrocytes from normal and degenerate articular cartilage is essential to understanding and dealing with pathophysiological changes that occur in joint disorders such as arthritis. Membrane transport systems regulate cell shape [7], cell volume [8], intracellular pH [9], intracellular signaling [10] and transepithelial transport [11]. In chondrocytes the extracellular ionic and osmotic environment also regulates the synthesis of extracellular matrix macromolecules [3]. The mechanical performance of cartilage relies on the biochemical properties of matrix macromolecules and any alterations to the ionic and osmotic extracellular environment of chondrocytes in turn influence the volume, intracellular pH and ionic content of the cells [12]. These changes in turn modify the synthesis and degradation of extracellular matrix macromolecules [13]. Physiological ion homeostasis is fundamental to the routine functioning of cartilage and the factors that control the integrity of this highly evolved and specialized tissue. Therefore, membrane transporters may prove suitable therapeutic targets in treating joint disorders in the future.

Na^+^, K^+^-ATPase is an important regulator of intracellular electrolyte levels in most mammalian cells [14]. It is a Mg^2+^-dependent transport pump responsible for maintaining the low intracellular Na^+^:K^+^ ratio that is essential for cell homeostasis and physiological function. It catalyzes the active uptake of K^+^ and extrusion of Na^+^ at the expense of hydrolyzing ATP with a stoichiometry of 3 Na^+^ for 2 K^+^. The active form of Na^+^, K^+^-ATPase is an integral membrane protein complex composed of three non-covalently attached subunits; a 110 kDa catalytic α subunit, a 45–55 kDa glycosylated β subunit and a 10 kDa proteolipid γ subunit [15,16]. Four α isoforms encoded by different genes have been identified which are ~85% identical at the protein level [17–19]. The β subunit also exists as four isoforms; three isoforms belong to Na^+^, K^+^-ATPase [20–22]. The fourth β isoform, β4, may function as an interchangeable component of the Na^+^, K^+^-ATPase and the non-gastric P-type H^+^, K^+^-ATPase but only in skeletal and cardiac muscle [23,24].

Differences in kinetic properties between Na^+^, K^+^-ATPase α isoforms have implications for Na^+^ and K^+^ transport rates and hence for Na^+^ dependent uptake of nutrients including amino acids, sugars and other vital nutrients [14]. The α subunit isoforms have shown significantly different affinities for Na^+^, K^+^, ATP and ouabain when expressed in HeLa cells and sf-9 insect cells (for a review see [14]). In addition the β isoforms alter the ion affinity of individual α subunits in α-β complexes [15].

Earlier work in our laboratories has revealed that primary and *in situ* chondrocytes abundantly express Na^+^, K^+^-ATPase (1.75 × 10^5^ sites per chondrocyte; [25,26]). Expression of Na^+^, K^+^-ATPase is sensitive to the extracellular ionic and osmotic environment within the extracellular matrix and *in vitro* [27,28]. We have also shown that Na^+^, K^+^-ATPase exists as multiple isozyme variants in bovine cartilage [25] and human cartilage [29]. The expression of three α (α1, α2, α3) and three β (β1, β2, β3) subunit isoforms in human cartilage indicates that up to nine different isozymes could be formed in this tissue [29]. Existence of multiple Na^+^, K^+^-ATPase isozymes implies the requirement for a finely tuned but varied ‘sodium pump’ for the specialized handling of transmembrane cation gradients. The aim of this study was to investigate the expression of Na^+^, K^+^-ATPase in a human chondrocyte cell line (C-20/A4) using a panel of well-characterized antibodies and a combination of immunological and biochemical techniques.

## 2. Results and Discussion

### 2.1. Characterization of the α Isoform Specific Antibodies

**Figure 1:**
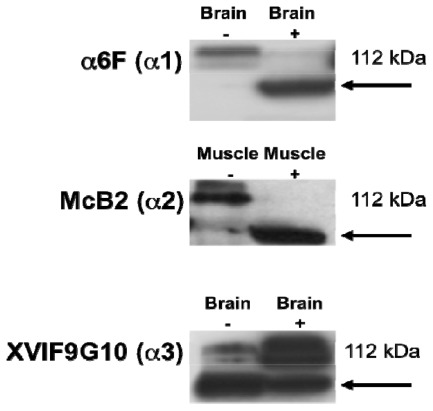
Western blots showing expression of Na^+^, K^+^-ATPase α subunit isoforms in human brain and skeletal muscle (positive control tissues); α1, α2 and α3 (112 kDa). + or − refer to samples being heated or not heated before electrophoresis. The data presented confirms that the α6F, McB2 and XVIF9G10 monoclonal antibodies recognize their target isoforms (α1, α2 and α3, respectively) in human brain and skeletal muscle.

Selected antibodies against the α1, α2 and α3 isoforms (α6F, McB2 and XVIF9G10 monoclonal antibodies) were characterized by western blotting to confirm their cross-reactivity with their respective protein targets in human brain and skeletal muscle (Figure 1).

### 2.2. Western Blotting of α and β Isoforms in C-20/A4 Cells

**Figure 2:**
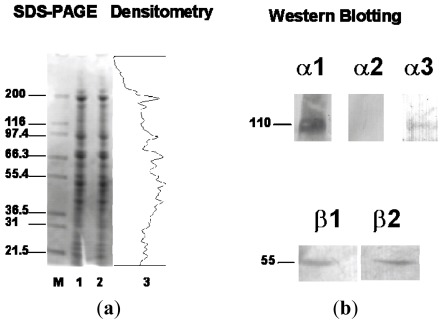

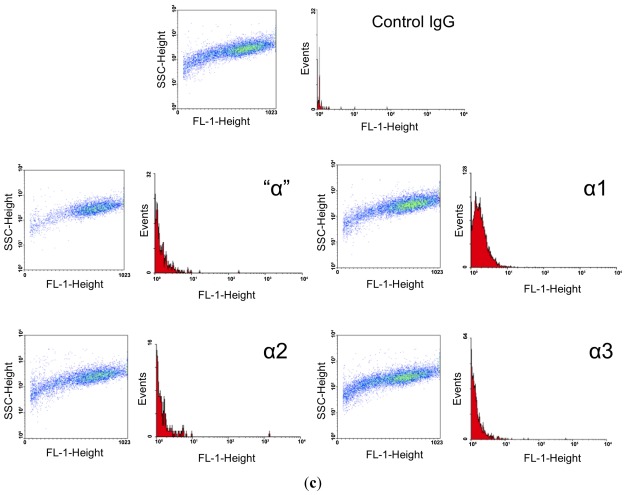
(**a**) Electrophoretic separation of human chondrocyte microsomes using Coomassie blue stained NuPAGE polyacrylamide gel. Molecular weight markers are shown in kDa (lane M) together with human chondrocyte plasma membrane enriched microsomal fractions (lanes 1 and 2). Lane 3 is a representative densitometric scan of the membrane proteins resolved in lane 2 and shows that most of the membrane proteins expressed in human chondrocyte-like cells are between 30 and 200 kDa markers with large aggregate peaks around 200, 95, 68 and 50 kDa. (**b**) Western blots showing expression of Na^+^, K^+^-ATPase isoforms in human chondrocyte-like cells; α1 and α3 (110 kDa), β1 and β2 (55 kDa) and the absence of α2. (**c**) FACS analysis of chondrocytes immunostained with monoclonal antibodies to the α subunit of Na^+^, K^+^-ATPase. In each panel the density plot on the left shows the size distribution of the cells analyzed and the histogram on the right shows the FITC positive cells as a frequency distribution. The highest FITC staining was observed with the α1 specific antibody followed by the pan α antibody. The lowest staining was observed with the non-immune control anti-mouse IgG followed by α2 and α3 specific antibodies.

The expression of Na^+^, K^+^-ATPase isoforms in plasma membrane enriched microsomes of C-20/A4 cells was investigated by western blotting. Membrane proteins were separated by SDS-PAGE in 4–12% NuPAGE gradient gels (Figure 2A). Membrane proteins were then blotted onto nitrocellulose membranes and blots incubated with isoform specific antibodies. Western blot analysis clearly established the presence of α1, α3, β1 and β2. Antibodies to α1 and α3 satisfactorily cross-reacted with protein bands between the 97.4 and 116 kDa markers (Figure 2B). As expected, the ubiquitous α1 isoform was found in higher quantities compared to α3 on the alkaline phosphatase-conjugated antibody-probed membranes. However, there was no evidence for α2 expression in chondrocytes. The western blots produced detectable bands within 10–15 min suggesting a low abundance of plasma membrane Na^+^, K^+^-ATPase in chondrocyte-like cells. The high specificity of the antibodies for their respective antigens in brain homogenates has previously been demonstrated using western blotting [25,26,30]. Due to the lack of specific antibodies to human α4 and β3, the expression of these isoforms was not investigated at the protein level.

### 2.3. FACS Analysis

FACS analysis demonstrated a relatively high level of α1 expression (67% positive cells) compared to α2 and α3 (1.14% and 5.28%, respectively; Table 1) in human chondrocyte-like cells. Autofluorescence was negligible, as was binding of non-immune mouse serum (Figure 2C). Expression levels of β1 and β2 isoforms were also found to be relatively high in these cells (79.49% and 87.19% respectively; see Table 1). Overall, the immunohistochemical, immunofluorescence, FACS and Western blot results presented in this paper suggest that although the α1, β1 and β2 isoforms are the most abundant isoform proteins expressed, lower, but nevertheless detectable levels of α3 protein present in human chondrocyte-like cells.

### 2.4. Immunofluorescence and Immunocytochemistry

**Figure 3:**
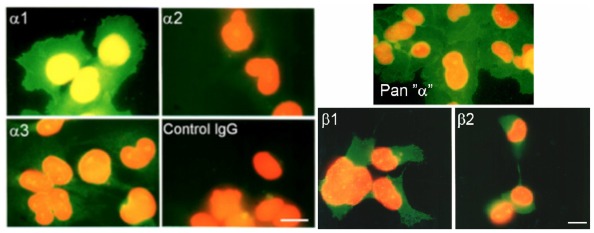
Indirect immunofluorescence localization of α and β isoforms of Na^+^, K^+^-ATPase in human chondrocyte-like cells attached to poly-l-lysine coated slides. Cells were probed with α and β isoform specific antibodies and secondary Fluorescein-conjugated IgG. Nuclei were counterstained red with propidium iodide. Bar indicates 10 μm.

**Figure 4:**
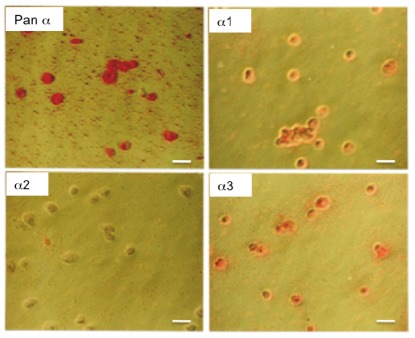
Immunohistochemical detection of Na^+^, K^+^-ATPase α isoforms in human chondrocytes using alkaline phosphatase-labeled anti-mouse and anti-rabbit secondary antibodies. Detection was by Fast Red TR/Napthol AS-MX phosphate (4-chloro-2-methylbenzenediazonium/3-Hydroxy-2-naphtoic acid 2,4-dimethylanilide phosphate). The following antibodies were used: pan α antibody, α1 (α 6F) antibody, α2 (McB2) antibody and α3 (XVIF9G10) antibody. Original magnifications in each panel × 400. Bar indicates 10 μm.

Immunocytochemical staining confirmed the presence of α1 and lower levels of α3 (Figures 3 and 4). The α1 isoform was found in abundance as expected; the monoclonal antibodies α620 and α6F, which had both reacted strongly on Western blots, also worked well in immunofluorescence experiments. The β1 and β2 subunit isoforms were also detected in roughly equal proportions (Figures 3 and 4). Immunoreactive cells exhibited strong alkaline phosphatase staining when probed with the pan α monoclonal antibody (mAb 9A7) and the α1 specific antibody; no α2 staining was observed but the α3 antibodies produced some immunostaining (Figures 3 and 4). Conventional immunofluorescence micrographs revealed strong expression of α1 on the plasma membrane. The pan α monoclonal antibody (mAb 9A7) produced the most intense immunofluorescence staining possibly due to the combined signal from α1 and α3 isoforms (Figure 3).

Recent studies of isolated bovine chondrocytes in our laboratory have revealed a high plasma membrane Na^+^, K^+^-ATPase density for these relatively small cells [25]. In addition to plasma membrane Na^+^, K^+^-ATPase, a large component is localized in intracellular membranes [32]. In bovine cartilage, the density of Na^+^, K^+^-ATPase correlates with matrix glycosaminoglycan concentrations and hence with tissue Na^+^ [27] suggesting a feedback mechanism for the regulation of Na^+^, K^+^-ATPase abundance by matrix proteoglycans. Molecular studies in bovine chondrocytes have revealed two α (α1 and α3) and two β (β1 and β2) Na^+^, K^+^-ATPase isoforms [25]. These early studies suffered from a number of limitations. First, they were completed before the cloning and identification of the α4 and β3 isoforms. Second, some of the antibodies used in the early studies were obtained from commercial sources and consequently were not sufficiently well characterized. Recent studies of Na^+^, K^+^-ATPase isoform expression in articular chondrocytes in human cartilage explants have resulted in a more consistent, but significantly more complicated description of isoform composition; 3 α (α1, α2 and α3) and 2 β isoforms (β1 and β2) are expressed in addition to several other key Na^+^ dependent transport systems [29,33].

The evidence for α3 expression in cartilage has gained considerable support since it was first proposed [25,26]. The well-characterized α3 specific monoclonal antibody XVIF9-G10 has been used successfully to demonstrate α3 expression in brain and heart [30] and articular cartilage [25,26,29]. Another well-characterized monoclonal antibody, McBX3, has been used successfully to demonstrate α3 expression in human articular cartilage [29]. There is evidence that the α3 subunit is post-translationally modified at the site of McBX3 binding in several tissues and this modification abolishes the affinity of McBX3 for its unique epitope on the α3 protein [34]. The fact that McBX3 is able to bind α3 in cartilage clearly validates that the α3 isoform expressed in cartilage is similar to the neuronal α3 isoform [29].

The expression of α3 may be related to the unusual ionic environment of chondrocytes [3]. In terms of their affinity for Na^+^, the α isoforms exhibit significantly different kinetic properties; the affinity of α3 for intracellular Na^+^ is orders of magnitude lower than α1 isoform (reviewed in [14,15]). It is therefore possible that chondrocyte Na^+^, K^+^-ATPase isozymes containing α3 subunits fulfill the vital physiological function of controlling intracellular Na^+^ during joint loading or other scenarios in which rapid changes occur to the intracellular Na^+^:K^+^ ratio [3]. Recent observations in isolated chondrocytes suggest that the α3 subunit may be preferentially upregulated along with the α1 subunit following long-term changes in extracellular Na^+^ resulting in steeper transmembrane Na^+^ gradients [32]. Chronic exposure of chondrocytes to high extracellular Na^+^ results in accumulation of α1 and α3 isoforms on the plasma membrane of chondrocytes and also in subcellular compartments [28]. Thus α3 with its low affinity for Na^+^ working in parallel with α1 isoform may allow chondrocytes to respond more effectively to physiological changes in intracellular Na^+^ that occur *in vivo* under load. Furthermore, the high affinity of α3 for ouabain raises the possibility of independent regulation of Na^+^, K^+^-ATPase isozymes by endogenous ouabain-like compounds.

The apparent lack of α2 expression in human chondrocyte-like cells is worthy of further discussion. Studies on isolated bovine articular chondrocytes have confirmed the absence of α2 expression [25,26]. Indeed, the predominant α isoforms expressed in isolated chondrocytes are α1 and α3, although α1 appears to be more abundant than α3 [25,26]. However, bovine and human articular chondrocytes *in situ* appear to express the α2 isoform [25,26,29]. The observation that α2 immunoreactivity is lost in isolated cells suggests that expression of this isoform is under hormonal regulation in cartilage. The lack of α2 in human chondrocyte-like cells supports this notion. In adipocytes (close relatives of chondrocytes) the α2 isoform is highly insulin sensitive in terms of its expression, regulation, function and cell surface recruitment [35,36]. It is possible that expression of α2 and possibly also other α isoforms is under hormonal control by insulin or IGF-1 (insulin like growth factor), a dominant anabolic mediator in cartilage.

The existence and variability of β isoforms in human chondrocytes also raises important issues that need to be addressed. Abundant expression of β1 in chondrocytes is not surprising given its ubiquitous tissue expression [20,21]. However, expression of β2 and β3 isoforms in chondrocytes, in addition to the β1 isoform, was surprising. The β2 isoform, also known as AMOG (adhesion molecule on glia) [37], is expressed in a number of tissues but mainly in neural tissues [38]. The β2 subunit is important for the development of the central nervous system [37,39,40] and mice deficient for AMOG/β2 exhibit neuronal [41].

The β3 isoform also appears to be expressed in human chondrocytes *in situ* [29] and human chondrocyte-like cells (this study). The β isoforms are glycoproteins displaying branched oligosaccharides to the extracellular environment. Since glycoproteins have been implicated in cell recognition and cell signaling, it may be possible that multiple β isoforms allow chondrocytes to detect changes in their extracellular environment. The α subunit of Na^+^, K^+^-ATPase has long been known to bind various intracellular cytoskeletal proteins including ankyrin [42,43] and actin [44,45]. β subunit isoforms may play a role in the biogenesis of chondrocyte Na^+^, K^+^-ATPase or modulating the longevity and intracellular trafficking of Na^+^, K^+^-ATPase complexes [14].

## 3. Experimental Section

### 3.1. Chemicals, Tissue Culture and Molecular Biology Kits

Chemicals were purchased from Sigma/Aldrich (Poole, UK). Chemicals used in molecular biology experiments were molecular biology grade and those used in cell culture and gel electrophoresis/ Western blotting procedures were tissue culture and electrophoresis grade respectively. Fetal bovine serum was obtained from Life Sciences (Paisley, Scotland) and Sigma/Aldrich. Molecular biology kits were obtained from BioGene Ltd (Kimbolton, UK) and Advanced Biotechnologies (Epsom, UK). Electrophoresis reagents were obtained from Novex (San Diego, USA). Oligonucleotide primers were obtained from Genosys Biotechnologies (Cambridgeshire, UK) and Genset SA Europe (Paris, France) affiliated with Helena Biosciences (Sunderland, UK). Thin-walled PCR tubes were purchased from Perkin Elmer (Norwalk, USA).

### 3.2. Human Tissues

Samples of human brain and skeletal muscle were used as positive control tissues for western blot characterization of the monoclonal antibodies to the α1, α2, α3 isoforms of the Na^+^, K^+^-ATPase. Tissues were obtained from the University Hospital of Universidad de La Laguna with local Ethics Committee approval. Plasma membrane enriched microsomes were prepared as described for C-20/A4 cells below.

### 3.3. Antibodies

Well characterized non-commercial monoclonal and polyclonal antibodies to the α1, α2, α3, β1 and β2 subunits of the Na^+^, K^+^-ATPase were selected based on our previous observations and information available in the literature. The overriding priority in our selection procedure was suitability for use in human cells. The antibodies employed in this study and information and references pertaining to their specificity are shown in Table 2. Specific antibodies are currently available against three α (α1, α2 and α3) and two β isoforms (β1 and β2). Although polyclonal antibodies against the β3 subunit are also available, these are not suitable for studies on human cells as they only recognize the β3 subunit of rat and mouse [46]. Secondary antibodies used were previously pre-adsorbed with human serum proteins by the manufacturer. Rhodamine-conjugated anti-rabbit IgG (H + L) pre-adsorbed with human, mouse and rat serum proteins were purchased from Jackson ImmunoResearch laboratories Inc (PA, USA). Fluorescein-conjugated anti-mouse IgG was obtained from Dako Ltd., (Cambridge, UK). Alkaline phosphatase-conjugated anti-mouse and anti-rabbit-IgG was obtained from Sigma/Aldrich.

### 3.4. Human Chondrocyte Cell Line

For all the experiments described a human chondrocyte-like cell line designated C-20/A4 was used [55]. The cells were grown in 75 cm^3^ flasks in Dulbecco’s modified Eagle’s medium with 1000 mg glucose/L, l-glutamine, NaHCO_3_ and pyridoxine HCl (Sigma D-6046) supplemented with 5% fetal calf serum (Gibco BRL 10108-157 or Sigma F-2442) and 1% antibiotic/antimycotic solution (Sigma A-5955) in a humidified incubator (95% air, 5% CO_2_). Cells from passages 12–24 were used in this study. In all experiments described, the cells were used when 95% confluent and 95% viable as determined by Trypan blue dye exclusion assays. The cells used in some immunological experiments were grown on poly-l-lysine coated slides. The morphology of the cells and the viability was not affected.

### 3.5. Preparation of Plasma Membrane Enriched Microsomes

C-20/A4 were lysed in a glass-teflon homogenizer using a homogenization buffer consisting of 50 mM Tris-HCl, 1 mM ethylenediamine-tetraacetic acid (EDTA), 1 mM phenylmethylsulphonyl fluoride (PMSF), and 1 mg/mL of each of the protease inhibitors aprotinin, leupeptin and pepstatin (to prevent non-specific proteolytic degradation). The lysate was centrifuged at 100,000 g for 35 min to obtain a fraction enriched in plasma membranes. Protein assays were performed using a Bio-Rad kit.

### 3.6. Gel Electrophoresis and Western Blotting

C-20/A4 membranes were subjected to electrophoresis using pre-cast Novex NuPAGE™ Bis-Tris-HCl buffered (pH 6.4) gels in a Novex X-CELL II™ unit. Assayed protein samples were diluted in 4× sample buffer (1.17 M sucrose, 563 mM Tris Base, 423 mM Tris HCl, 278 mM SDS, 2.05 mM EDTA, 0.88 mM Serva Blue G250, 0.7 mM phenol red and ultrapure water). For the majority of experiments described 1.0 mm thick 4–12% gradient gels were used for optimum electrophoretic resolution. A MOPS (3-(*N*-morpholino) propane sulfonic acid) SDS (sodium dodecyl sulfate) running buffer was used. Electrophoretic transfer to nitrocellulose membranes (Hybond ECL, Amersham-Pharmacia, Bucks, UK) was performed in the same unit using a blot transfer module. The transfer buffer consisted of Bicine, Bis-Tris and EDTA.

### 3.7. Cell Fixation and Immunolabeling

C-20/A4 cells were allowed to attach to poly-l-lysine pre-coated slides (BDH, UK). Cells were fixed in ice-cold methanol for 10 min at −20 °C. After removal from the fixative the cells were washed once with PBS. Subsequently the cells were washed 3× with PBS. Non-specific binding was blocked by incubating the cells with PBS containing 10% normal goat serum (NGS; Sigma) for 1 h at room temperature. Primary α and β-isoform specific antibodies (see Table 2) were diluted in PBS containing 1% NGS and incubated with C-20/A4 cells for 2 h at room temperature. The cells were then washed 4× in PBS, and incubated with FITC-conjugated and TRITC-conjugated anti-mouse or anti-rabbit IgG (Sigma) in PBS containing 1% NGS (2 h at room temperature). The cells were finally washed 4× with PBS and mounted in mounting medium (Vectashield, Vector Laboratories Burlingame CA). In some immunocytochemical experiments, alkaline phosphatase- labeled anti-mouse and anti-rabbit secondary antibodies were used. For localization of alkaline phoshophatase activity Sigma Fast Red TR/Napthol AS-MX phosphate (4-chloro-2-methylbenzenediazonium/3-Hydroxy-2-naphtoic acid 2,4-dimethylanilide phosphate) tablets were used.

### 3.8. Immunofluorescence Microscopy

Chondrocytes probed with α and β isoform specific antibodies using procedures described above were examined using a Zeiss fluorescence microscope fitted with an oil immersion objective (100×) and a MC 100 SPOT 35 mm microscope camera with automatic exposure control.

### 3.9. FACS Analysis

C-20/A4 chondrocytes grown to confluence as monolayer cultures were removed from culture flasks by treatment with trypsin EDTA (Life Technologies, UK), washed three times with PBS and fixed in 3.7% neutral buffered paraformaldehyde (Sigma, UK) in PBS for 10 min at 37 °C. The cells were washed three times with PBS and aliquots of 10^−5^ cells/100 mL PBS were placed into 1.5 mL eppendorf tubes, spun down (2000 rpm, 5 min) and the cell pellets were incubated with 100 mL aliquots of α subunit specific monoclonal antibodies (pan α, α6F, McB2 and McBX3; see Table 2 for dilutions used) at 4 °C overnight. Negative controls were incubated with 100 mL of a non-immune mouse IgG (DAKO, UK). Following three washes with PBS, the cells were incubated with 100 mL FITC-conjugated goat anti-mouse IgG diluted 1:100 (Sigma, UK) in PBS at 4 °C for 1 h in the dark. After three further washes in PBS, the cells were resuspended in 0.5 mL PBS and analyzed on a Becton-Dickinson FACScan. Plot profiles and histograms were generated using WinMDI flow cytometry software [31]. Statistical analysis was also performed using WinMDI.

## 4. Conclusions

Chondrocytes are routinely exposed to a variable ionic and osmotic environment in cartilage matrix. Maintaining an optimal Na^+^:K^+^ ratio is essential for intracellular homeostasis and for the synthesis of a mechanically resilient extracellular matrix. This study confirms that Na^+^, K^+^-ATPase in chondrocyte-like cells exists in a variety of isozyme combinations. Different combinations of α and β isoforms have been expressed in human and insect cell models and results suggest that experimental exchange of isoforms modifies pump kinetics (affinities for ions, ATP and ouabain) and functional properties of the enzyme complex (reviewed in [14]). The existence of multiple forms of Na^+^, K^+^-ATPase in human chondrocytes *in situ* and the expression of the same isoforms in the immortalized human chondrocyte-like cells suggest that the C-20/A4 exposed to different extracellular environments, anti-inflammatory drugs, growth factors, hormones and cytokines. This approach would help determine the contribution of ion transport to cellular repair processes that may be initiated following extracellular matrix degradation in pathologies of articular cartilage. It would be premature to speculate upon any direct involvement of Na^+^, K^+^-ATPase in pathologies of articular cartilage. Na^+^, K^+^-ATPase and other transport systems may prove to be indirectly involved in the cellular adaptation to extracellular matrix changes that occur in arthritis.

This study confirms previous observations in isolated bovine chondrocytes [26] and human chondrocytes [29] supporting a crucial role for Na^+^, K^+^-ATPase in chondrocyte homeostasis and cartilage maintenance. Now that the molecular composition of Na^+^, K^+^-ATPase and the relative abundance of its subunit isoforms’ expression is established, the next challenge is to evaluate the effect of cartilage specific anabolic growth factors and catabolic cytokines on the abundance and activity of Na^+^, K^+^-ATPase as a system and the expression of its subunit constituents. This objective will only be accomplished using a suitable cell-line instead of freshly isolated cells. The C-20/A4 cell-line is a convenient and easily accessible chondrocyte model, which can be grown under standard conditions in different laboratories and studied independently.

## Figures and Tables

**Table 1:** Quantitative analysis of Na^+^, K^+^-ATPase isoform distribution in C-20/A4 cells. Chondrocytes immunostained using α subunit specific antibodies and secondary FITC-conjugated anti-mouse IgG were examined by FACScan and the data analyzed using WinMDI 2.8 flow cytometry software [31].

Antigen	Number of Cells Counted	Number of FITC Positive Cells	% FITC Labeling	Mean Fluorescence Intensity
α1	6800	4568	67.18	19.2
α2	3963	45	1.14	16.5
α3	5904	312	5.28	16.1
β1	195	155	79.49	36.18
β2	3287	2866	87.19	45.83

**Table 2:** Isoform specific monoclonal and polyclonal antibodies employed in this study.

Antibody	Isoform	Working Dilution	Source	Reference
mAb 9A7 (pan α monoclonal)	α isoforms	1:100	M. Takahashi	[47]
α5 § (monoclonal)	α isoforms	Neat Supernatant	D.M. Fambrough and DSHB *	[48]
α6F (monoclonal)	α1	Neat Supernatant	D.M. Fambrough and DSHB *	[49]
α620 (polyclonal)	α1	1:100–1:200	M. Caplan	[50,51]
McB2 (monoclonal)	α2	Neat Supernatant	K.J. Sweadner	[52]
McBX3 (monoclonal)	α3	Neat Supernatant	K.J. Sweadner	[30,46]
XVIF9G10 (monoclonal)	α3	Neat Supernatant	K.J. Sweadner	[30,46]
SpETb1 (polyclonal)	β1	1:200–1:400	P. Martín-Vasallo	[53]
SpETb2 (polyclonal)	β2	1:200–1:400	P. Martín-Vasallo	[53]

* The α5 and α6F monoclonal antibodies were developed by D. Fambrough were obtained from the Developmental Studies Hybridoma Bank (DSHB [54]) developed under the auspices of the NICHD and maintained by The University of Iowa, Department of Biological Sciences, Iowa City, IA 52242, USA.

§ The α5 monoclonal broadly recognizes Na^+^, K^+^-ATPase α subunits of avian, mammalian and insect species. There are 4 known α isoforms and the designation “α5” does not indicate the existence of a fifth Na^+^, K^+^-ATPase α isoform.
